# Duplicate-Aware Internal Validation of Machine-Learning Models for Classifying a Tanita BIA-Derived High-Adiposity Phenotype Using Simple Anthropometric Predictors

**DOI:** 10.3390/jcm15166164

**Published:** 2026-08-08

**Authors:** Rukiye Çiftçi, İpek Atik, Neşe Bülbül, Özgür Eken, Monira I. Aldhahi

**Affiliations:** 1 Department of Anatomy, Medical Faculty, Gaziantep Islam Science and Technology University, Gaziantep 27010, Türkiye; rukiye.ciftci@gibtu.edu.tr; 2 Department of Electrical Electronics Engineering, Gaziantep Islam Science and Technology University, Gaziantep 27010, Türkiye; ipek.atik@gibtu.edu.tr; 3 Department of Endocrinology and Metabolism, Gaziantep Islam Science and Technology University, Gaziantep 27010, Türkiye; nese.bulbul@gibtu.edu.tr; 4 Department of Physical Education and Sport Teaching, Faculty of Sports Sciences, İnönü University, Malatya 44280, Türkiye; ozgur.eken@inonu.edu.tr; 5 Department of Rehabilitation Sciences, College of Health and Rehabilitation Sciences, Princess Nourah bint Abdulrahman University, P.O. Box 84428, Riyadh 11671, Saudi Arabia

**Keywords:** adiposity, bioelectrical impedance analysis, machine learning, data leakage, duplicate-aware validation, calibration, SHAP, hypertension status

## Abstract

**Background/Objectives:** Anthropometric machine-learning models may approximate body-composition classifications, but performance can be inflated by inconsistent preprocessing, non-independent validation records, and incomplete calibration reporting. This study evaluated a sex-specific bioelectrical impedance analysis (BIA)-defined high-adiposity phenotype using simple anthropometric variables in adults with and without hypertension. **Methods:** Of 583 prespecified records, 11 with invalid placeholder-coded values in required anthropometric or body-composition fields were excluded, leaving 572 participants (385 normotensive and 187 hypertensive). A high-adiposity phenotype was defined as BIA-derived body fat ≥ 25% in males or ≥35% in females. Predictors were sex, height, body weight, waist circumference, and hypertension status; body mass index and body-fat percentage were excluded. Eight algorithms were assessed using 10 repetitions of stratified five-fold group cross-validation, with identical predictor profiles kept within the same fold. Continuous predictors were standardized within training folds. Performance was estimated from averaged out-of-fold probabilities with 2000 stratified bootstrap confidence intervals, calibration measures, and SHAP analysis. **Results:** A high-adiposity phenotype was present in 441 participants (77.1%). Random forest achieved the highest discrimination (ROC AUC = 0.959, PR AUC = 0.980). Gradient boosting provided the strongest threshold-dependent performance (accuracy = 0.937, balanced accuracy = 0.895, sensitivity = 0.973, specificity = 0.817, precision = 0.947, F1 score = 0.960, MCC = 0.817) and the lowest Brier score (0.057), but its calibration slope was 0.462, indicating overconfident probabilities. SHAP analysis identified waist circumference as the largest attribution within the fitted gradient-boosting model; this result must be interpreted jointly with the ablation analysis because sex, height, and body weight are inputs to the proprietary Tanita equation. **Conclusions:** Simple anthropometric variables classified the prespecified Tanita BIA-derived high-adiposity threshold with strong internal performance after duplicate-aware validation. Sex, height, and body weight alone achieved a ROC AUC of 0.920; adding waist circumference produced a small and uncertain increase in discrimination (ΔROC AUC = 0.0054, 95% CI −0.0060 to 0.0153), whereas hypertension status added a negligible value. The findings represent internal validation of a device-defined outcome, not prediction of an independent biological reference, and require external validation against criterion body-composition methods before clinical application.

## 1. Introduction

Obesity and excess adiposity are major contributors to cardiometabolic disease, disability, and premature mortality. A pooled analysis of 3663 population-representative studies documented a marked global increase in obesity between 1990 and 2022 [1]. Contemporary diagnostic guidance further distinguishes excess adiposity from clinical obesity, which requires evidence of obesity-related organ dysfunction or meaningful functional limitation rather than reliance on body size alone [2]. This distinction makes accurate characterization of adiposity clinically important while also requiring careful terminology when a study uses an operational body-composition threshold.

Body mass index (BMI) remains the most widely used screening measure because it is inexpensive and highly reproducible, but it cannot distinguish fat mass from fat-free mass or describe regional fat distribution. In a large analysis of adults with dual-energy X-ray absorptiometry (DXA) data from the United States and Korea, the relationship of BMI with percentage of body fat and fat mass index varied by age, sex, and race–ethnicity and weakened in older adults and in the Korean population [3]. Central adiposity provides complementary risk information. In approximately 500,000 UK Biobank participants, measures of central adiposity remained strongly associated with incident heart failure and added information beyond general adiposity [4]. These observations support the inclusion of waist circumference when the objective is to characterize adiposity-related risk using measurements that are feasible in routine settings. Sex is also a relevant source of heterogeneity. Imaging-based data demonstrate differences in total and regional adiposity between women and men, whereas longitudinal cohort data show distinct life-course blood-pressure trajectories by sex [5,6].

Bioelectrical impedance analysis (BIA) is non-invasive, rapid, and comparatively inexpensive, but it estimates body composition indirectly through device- and equation-specific models. Recent validation studies against DXA have reinforced that agreement depends on the population, sex distribution, body size, measurement protocol, and the equations embedded in the instrument. New BIA equations developed in a heterogeneous Caucasian sample improved fat-mass-percentage estimation relative to non-specific equations [7]. In young adults, BIA and anthropometric estimates showed systematic and individual-level differences from DXA, with sex and hydration-related factors affecting agreement [8]. A subsequent segmental analysis similarly showed that the validity of BIA and low-cost anthropometric models varied by body region and outcome [9]. BIA-derived body fat should, therefore, be treated as a device-dependent estimate rather than an interchangeable substitute for a criterion body-composition method.

Machine-learning methods can capture nonlinear associations and interactions among anthropometric variables and have increasingly been evaluated for obesity and cardiometabolic classification. In a 2025 study of 5372 adults, random forest achieved an area under the receiver operating characteristic curve of 0.947 for obesity-level classification using anthropometric and BIA-derived indices; fat mass index, fat-free mass index, and BMI were the most influential predictors [10]. Body-composition variables have also shown strong performance in machine-learning models for hypertension status classification [11]. These findings demonstrate predictive feasibility, but they also highlight a methodological concern. When predictors and outcomes originate from the same body-composition system or include variables used to define the target, apparent performance may partly reflect circularity or target leakage.

Other sources of optimistic performance include preprocessing the full dataset before validation, selecting models on the same data used for performance estimation, and allowing duplicate or highly similar profiles to occur in both training and validation sets. Empirical work has shown that dependence-related data leakage can materially inflate predictive performance [12]. Moreover, prediction quality is multidimensional. Transparent model evaluation should include discrimination, threshold-dependent classification, calibration, overall prediction error, uncertainty, and clinically relevant consequences, rather than accuracy alone [13,14,15,16,17,18,19]. Feature-attribution methods such as SHAP can complement these metrics by identifying which variables drive individual and global predictions [20], but interpretability does not compensate for biased validation or an imperfect reference outcome.

The present study, therefore, aimed to evaluate eight machine-learning algorithms for classifying a prespecified Tanita BIA-derived high-adiposity phenotype using sex, height, body weight, waist circumference, and hypertension status while excluding BMI and BIA-derived body-fat percentage from the predictor set. Because sex, height, and body weight are also incorporated into the proprietary equation used by the BIA device to estimate body-fat percentage, the present analysis should be interpreted as evaluating the classification of a device-defined phenotype rather than an independent biological reference. Accordingly, the study focuses on methodological robustness through duplicate-aware validation, calibration assessment, uncertainty estimation, and model interpretability, while recognizing that external validation against criterion body-composition methods remains necessary.

## 2. Materials and Methods

### 2.1. Study Design, Setting, and Participants

This single-center cross-sectional prediction-model study included adults assessed at the Endocrinology Outpatient Clinic of Ersin Arslan Training and Research Hospital, Gaziantep, Türkiye. The study is reported in accordance with the STROBE principles for cross-sectional studies and the TRIPOD recommendations for prediction-model studies [13]. Participants with and without a diagnosis of hypertension who were evaluated between 15 July 2025 and 15 January 2026 were included in the study. The study was conducted in accordance with the principles of the Declaration of Helsinki. Sampling was convenience-based. Eligibility criteria in the source protocol were age 18–65 years, ambulatory status, no previous abdominal surgery, and no implanted medical device. Exclusion criteria were pregnancy, chronic kidney disease, cancer, and use of medications expected to substantially affect body composition, including systemic corticosteroids.

The study was approved by the Ethics Committee for Non-Interventional Clinical Research at Gaziantep Islam Science and Technology University (approval number 2025/644; approval date: 29 June 2025) and was conducted in accordance with the Declaration of Helsinki. Written informed consent was obtained from all participants. Age was not available in the analytic dataset and, therefore, could not be evaluated as a predictor. Because age is incorporated into the proprietary Tanita equation used to estimate body-fat percentage, its absence prevents direct assessment of the independent contribution of age and represents an additional source of potential incorporation bias.

Hypertension was defined as a previous physician diagnosis of hypertension documented in the participants’ medical records and/or the current use of antihypertensive medication. Participants without a documented diagnosis of hypertension and not receiving antihypertensive treatment were classified as normotensive. Hypertension status was, therefore, determined using established clinical records rather than a single blood pressure measurement obtained at the study visit.

No formal a priori sample-size calculation was performed because this study was a secondary analysis of an existing clinical dataset. All eligible participants with complete data for the required variables were included, yielding 572 participants (385 normotensive and 187 hypertensive). This sample supported descriptive internal comparison of the prespecified models. However, the smaller non-high-adiposity class and sparse sex-by-hypertension strata limited the precision of subgroup, calibration, and transportability assessments.

### 2.2. Anthropometric and Body-Composition Measurements

Measurements were performed between 08:00 and 11:00 after a minimum 8 h fast. Participants were instructed to avoid strenuous exercise, caffeine, and alcohol for 24 h before assessment. Height, body weight, and waist circumference were obtained using standardized procedures. Waist circumference was measured midway between the lowest rib and iliac crest with a flexible tape.

Body composition was assessed with a Tanita TBF-305 foot-to-foot BIA analyzer (Tanita Corporation, Tokyo, Japan). Participants stood for 10 min before measurement and were then assessed barefoot while remaining still. Before BIA estimation, sex, age, and height were entered into the analyzer. Body weight was measured directly by the device, and these inputs were used in the manufacturer’s proprietary equation to generate a body-fat percentage. No separate clothing-weight adjustment was documented in the de-identified analytic files. BMI was calculated as body weight in kilograms divided by height in meters squared. BIA values were treated as device-derived estimates rather than criterion measurements [7,8,9].

### 2.3. Data-Integrity Audit and Analytic Sample

The two prespecified primary data sheets contained 583 records: 392 normotensive and 191 hypertensive. Eleven records containing invalid placeholder-coded values in required anthropometric or body-composition variables were excluded before analysis: body-fat percentage was coded as 2 in 10 records (six normotensive and four hypertensive), and waist circumference was coded as 2 in one normotensive record. Seven normotensive and four hypertensive records were, therefore, excluded, yielding 572 complete records (385 normotensive and 187 hypertensive). All remaining records were retained; no imputation was performed. Variable-level exclusion counts are reported in Supplementary Table S1.

Because unique participant identifiers were not available in the source analytic files, exact predictor-profile duplication was audited before validation. The five predictor vectors yielded 480 unique profiles among 572 records, corresponding to 92 repeated profiles. Some identical profiles may represent distinct participants with rounded measurements. However, to prevent optimistic validation if any were duplicate entries, all records sharing an exact predictor profile were assigned to the same cross-validation fold. This conservative procedure addresses dependence-related leakage [12].

### 2.4. Outcome and Predictor Definitions

The operational outcome was a sex-specific BIA-defined high-adiposity phenotype: body-fat percentage ≥ 25% for males or ≥35% for females [21]. Because these cut points are conventional research thresholds and not a universally accepted diagnosis of clinical obesity, the outcome is described throughout as a threshold-defined high-adiposity phenotype [2,22].

The prespecified predictors were sex (female = 0, male = 1), height (cm), body weight (kg), waist circumference (cm), and hypertension status (no = 0, yes = 1). BMI and BIA-derived body-fat percentage were excluded from the predictor set. Height, body weight, and waist circumference were standardized within each training fold using the training-fold mean and standard deviation. Binary predictors were passed through without standardization. All preprocessing was implemented inside the model pipeline.

### 2.5. Machine-Learning Algorithms

Eight classifiers were compared using fixed, prespecified hyperparameters to avoid validation-set optimization: logistic regression (C = 1), random forest (200 trees, maximum depth = 10, minimum leaf size = 2), gradient boosting (200 estimators, maximum depth = 5, learning rate = 0.1), linear support vector machine (C = 1, probability estimation enabled), k-nearest neighbors (k = 5), decision tree (maximum depth = 5, minimum leaf size = 2), Gaussian naive Bayes, and AdaBoost (100 estimators, learning rate = 0.1). Analyses were performed using Python 3.12 with scikit-learn 1.6.1 [23], NumPy 2.2.6 [24], SciPy 1.15.3 [25], and SHAP 0.48.0 [20].

Hyperparameters were intentionally specified as a priori and held constant across all repeated cross-validation iterations to ensure reproducibility and to isolate the effect of the validation strategy rather than extensive model optimization. Consequently, comparative performance should be interpreted as reflecting these prespecified model configurations rather than globally optimized implementations. Accordingly, the primary objective was a methodological comparison under identical validation conditions rather than identification of the single best-performing classifier.

### 2.6. Duplicate-Aware Internal Validation

Primary internal validation used 10 repetitions of stratified 5-fold group cross-validation. Groups were defined by the exact five-predictor profile, so that identical profiles could not be divided between training and validation folds. Within each repetition, every participant received one out-of-fold probability; the 10 out-of-fold probabilities were then averaged for participant-level performance estimation. This design used all available observations for model development and validation while reducing the optimistic bias that can arise from a single random split [12,15,16].

To directly quantify the effect of profile grouping, a sensitivity analysis was also performed using conventional record-level repeated stratified five-fold cross-validation, in which identical predictor profiles were allowed to occur in different folds. The same preprocessing pipelines, model specifications, number of repetitions, and probability threshold were used. Performance estimates from conventional and duplicate-aware validation were compared using ROC AUC, F1 score, and Brier score. Differences were calculated as duplicate-aware grouped validation minus conventional record-level validation.

### 2.7. Performance and Statistical Analysis

Discrimination was quantified with the area under the receiver operating characteristic curve (ROC AUC) and area under the precision–recall curve (PR AUC). At the prespecified probability threshold of 0.50, the accuracy, balanced accuracy, sensitivity, specificity, precision, F1 score, and Matthews correlation coefficient (MCC) were calculated. Overall probabilistic accuracy was assessed with the Brier score. Calibration was evaluated using a calibration plot, calibration intercept, and calibration slope; an intercept of 0 and slope of 1 represent ideal calibration [17,18,19]. The primary analytic purpose was binary classification at the fixed 0.50 threshold rather than individualized absolute-risk estimation. No recalibration model was fitted; predicted probabilities were retained to compare discrimination, calibration, and overall probabilistic error across algorithms.

Two-sided 95% CIs were estimated using 2000 stratified participant-level bootstrap resamples of the aggregated out-of-fold predictions, separately resampling high-adiposity and non-high-adiposity records to preserve class counts. These CIs quantify uncertainty in the observed out-of-fold predictions but do not fully capture model-development variability. Because the objective was descriptive model comparison rather than formal null-hypothesis testing, no multiple-comparison testing was conducted. Participant characteristics are summarized with the mean ± standard deviation and standardized mean differences (SMDs); *p* values were intentionally not used to characterize baseline group differences. For continuous variables, SMD was calculated as the between-group mean difference divided by the pooled standard deviation. For binary variables, the difference in proportions was divided by the pooled Bernoulli standard deviation.

To quantify partial incorporation bias and the incremental predictive contribution of variables not directly entered into the BIA device, an additional ablation analysis was conducted using logistic regression as an interpretable benchmark. Three nested predictor sets were evaluated: (i) sex, height, and body weight; (ii) sex, height, body weight, and waist circumference; and (iii) the complete predictor set additionally including hypertension status. Each model was assessed using the same repeated duplicate-aware group cross-validation procedure applied in the primary analysis. Differences in ROC AUC, F1 score, and Brier score between nested models were calculated from paired participant-level out-of-fold predictions. Because the primary objective of the ablation analysis was descriptive comparison of the incremental predictive contribution of each predictor set, the nested models were compared using their observed performance estimates. A paired bootstrap confidence interval was additionally calculated for the change in ROC AUC to aid in the interpretation of the incremental discrimination provided by waist circumference.

Because the outcome was defined using sex-specific body-fat thresholds and the study population was markedly female predominant, an additional female-only sensitivity analysis was performed. All eight algorithms were evaluated among female participants using the same preprocessing procedures, fixed hyperparameters, repeated stratified five-fold duplicate-aware group cross-validation, probability threshold, and participant-level averaging of out-of-fold predictions as in the primary analysis. Male-specific model performance was not treated as a confirmatory analysis because of the limited number of male participants.

Because hypertension status was emphasized in the study framing, the aggregated out-of-fold predictions from the primary gradient-boosting model were additionally stratified by hypertension status. Within each stratum, ROC AUC, PR AUC, accuracy, balanced accuracy, sensitivity, specificity, F1 score, MCC, Brier score, and confusion-matrix counts were calculated. The model was not refitted separately within the subgroups. Rather, subgroup performance was assessed from the primary out-of-fold predictions to preserve the original validation design.

To assess whether complex machine-learning models provided meaningful value beyond simple anthropometric benchmarks, additional comparator models were evaluated using the same repeated duplicate-aware group cross-validation procedure. The benchmark models consisted of logistic regression using waist circumference alone, waist circumference-to-height ratio alone, body weight plus waist circumference, and the complete prespecified predictor set. Their performance was compared with random forest and gradient boosting using ROC AUC, PR AUC, accuracy, balanced accuracy, sensitivity, specificity, F1 score, MCC, and Brier score.

All reported performance metrics were calculated from the averaged out-of-fold predicted probabilities obtained across the ten repeated duplicate-aware cross-validation iterations.

### 2.8. Model Interpretation

Gradient boosting was selected for SHAP interpretation because it had the strongest threshold-based classification profile and the lowest Brier score. The final gradient-boosting model was fitted to the full analytic dataset after the internal-validation analysis. TreeExplainer SHAP values were used to summarize the direction and magnitude of the feature contributions [20]. The SHAP analysis was explanatory and did not constitute an additional validation procedure.

**Figure 1:**
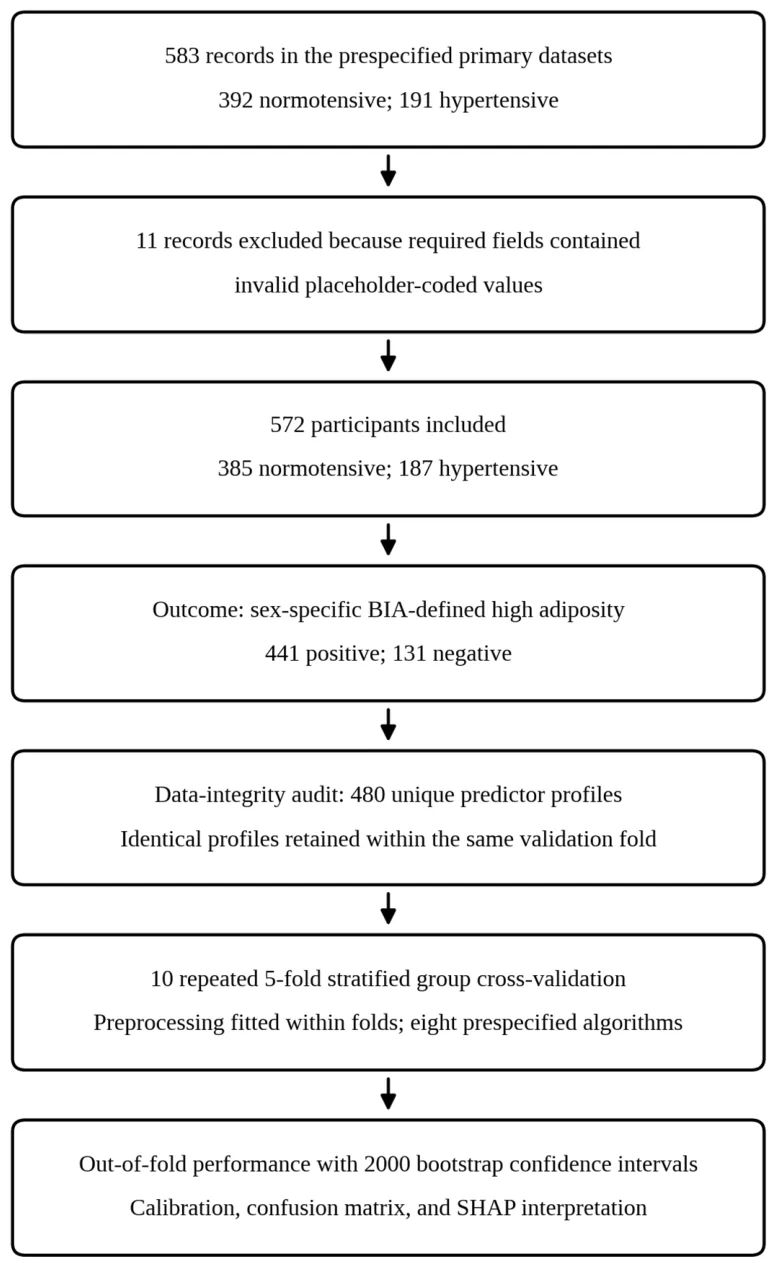
Participant flow, data-integrity audit, duplicate-aware validation, and performance evaluation workflow.

Figure 1 summarizes the complete analytic pathway. Of the 583 prespecified records, 11 were excluded because the required fields contained invalid placeholder-coded values, leaving 572 participants for analysis. The audit identified 480 unique predictor profiles, and all records sharing an identical profile were retained within the same validation fold. The figure, therefore, documents both the derivation of the analytic sample and the safeguards used to reduce dependence-related leakage before repeated cross-validation, bootstrap uncertainty estimation, calibration analysis, confusion-matrix assessment, and SHAP interpretation.

## 3. Results

### 3.1. Sample and Data Integrity

The final sample contained 572 participants, including 509 females and 63 males. The high-adiposity phenotype was identified in 441 participants (77.1%); 131 participants (22.9%) were below the prespecified threshold. The prevalence was 68.6% (264/385) in the normotensive group and 94.7% (177/187) in the hypertensive group. The hypertensive group contained only seven males (3.7%), compared with 56 males (14.5%) in the normotensive group. The duplicate audit identified 480 unique five-predictor profiles; the maximum frequency of an exact profile was five. Sex-specific descriptive counts showed that 406 of 509 females (79.8%) and 35 of 63 males (55.6%) met their respective sex-specific high-adiposity thresholds. Because the thresholds differed by sex and the sample was strongly female-predominant, these percentages are descriptive and should not be interpreted as an inferential between-sex comparison.

Table 1 indicates pronounced descriptive differences between the hypertension status groups. Compared with the normotensive participants, the hypertensive participants had a larger waist circumference (SMD = 1.133), BMI (SMD = 1.028), body-fat percentage (SMD = 0.899), and body weight (SMD = 0.738), together with a lower height (SMD = −0.717). The high-adiposity phenotype was also more prevalent in the hypertensive group (94.7% vs. 68.6%; SMD = 0.715). The greater female predominance in the hypertensive group (96.3% vs. 85.5%; SMD = 0.382) highlights the sex imbalance and supports a cautious interpretation of group-specific differences.

### 3.2. Duplicate-Aware Model Performance

Random forest achieved the highest discrimination, with a ROC AUC of 0.959 and a PR AUC of 0.980. Gradient boosting achieved the highest accuracy, balanced accuracy, F1 score, and MCC and had the lowest Brier score. At the prespecified 0.50 threshold, gradient boosting correctly classified 429 of 441 high-adiposity records and 107 of 131 records below the threshold, with 12 false negatives and 24 false positives. K-nearest neighbors also performed strongly, whereas Gaussian naive Bayes attained the highest specificity but substantially lower sensitivity.

Table 2 shows that the algorithms differed primarily in their discrimination and sensitivity–specificity balance. Under the prespecified hyperparameter settings, random forest achieved the highest ROC AUC and PR AUC, whereas gradient boosting achieved the highest balanced accuracy and F1 score. Decision tree and k-nearest neighbors also showed strong threshold-based performance. Gaussian naive Bayes attained the highest specificity but had substantially lower sensitivity, whereas AdaBoost preserved very high sensitivity at the expense of low specificity. Because hyperparameters were intentionally fixed before analysis, the observed ranking should be interpreted as a comparison under standardized validation conditions rather than as evidence that one algorithm is universally superior. Precision–recall curves for all evaluated classifiers are provided in Supplementary Figure S1.

Table 3 demonstrates differences in the overall prediction error and confusion-matrix patterns across the models. Gradient boosting achieved the highest accuracy and MCC and the lowest Brier score, making 36 total classification errors. Random forest made 46 errors. Decision tree made 47 errors, and k-nearest neighbors made 55 errors. Gaussian naive Bayes produced a comparatively large number of false negatives, whereas AdaBoost produced many false positives. Calibration performance is presented separately because accurate classification at a fixed threshold does not necessarily imply well-calibrated predicted probabilities. Despite its strong fixed-threshold classification performance, gradient boosting was not well-calibrated. The calibration slope of 0.462 indicates overconfident probabilities and limits probability-based interpretation without external validation and, if needed, recalibration.

Models were evaluated using 10 repetitions of stratified five-fold group cross-validation, with records sharing an identical five-predictor profile retained within the same fold. Participant-level predictions were obtained by averaging the 10 out-of-fold probabilities. The first model included variables entered directly into the Tanita device equation that were available in the analytic dataset. MCC, Matthews correlation coefficient; PR AUC, precision–recall area under the curve; ROC AUC, receiver operating characteristic area under the curve.

To quantify the potential influence of partial incorporation bias, an additional nested logistic-regression analysis was performed using the same duplicate-aware repeated group cross-validation procedure. As shown in Table 4, a model including only sex, height, and body weight achieved a ROC AUC of 0.920 and a PR AUC of 0.965, indicating that variables incorporated into the proprietary Tanita equation already provided substantial discriminatory ability. Adding waist circumference increased ROC AUC modestly to 0.925 and improved balanced accuracy, specificity, F1 score, and Brier score. The paired bootstrap estimate for the change in ROC AUC was 0.0054 (95% CI −0.0060 to 0.0153), so the discrimination gain was small and uncertain. Inclusion of hypertension status produced virtually no further improvement. These findings indicate that most observed discrimination originated from variables embedded in the device-derived reference outcome, while waist circumference contributed modest incremental information and hypertension status provided negligible incremental value.

To quantify the effect of duplicate-aware validation, all models were additionally evaluated using conventional record-level stratified cross-validation. Overall, the differences between conventional and duplicate-aware validation were modest, indicating limited optimism arising from repeated predictor profiles. The largest reduction in ROC AUC under duplicate-aware validation was observed for gradient boosting (ΔROC AUC = −0.008), followed by k-nearest neighbors, random forest, and AdaBoost (each ΔROC AUC = −0.003). Logistic regression, linear SVM, and decision tree showed virtually no change in discrimination, whereas Gaussian naive Bayes differed by only −0.001. Similar patterns were observed for the F1 score and Brier score, with the greatest deterioration in probabilistic accuracy occurring for gradient boosting (ΔBrier = +0.013). Overall, these findings indicate that duplicate-aware validation produced slightly more conservative performance estimates, particularly for the more flexible ensemble methods, while the overall ranking of the algorithms remained largely unchanged (Table 5).

#### 3.2.1. Incremental Contribution of Predictors Beyond the Tanita Device Equation

To quantify the potential influence of partial incorporation bias, an additional nested logistic-regression analysis was performed using the same duplicate-aware repeated group cross-validation procedure. Sex, height, and body weight alone achieved an ROC AUC of 0.920 and a PR AUC of 0.965. Adding waist circumference produced modest improvements in threshold-dependent performance and Brier score, but the paired bootstrap confidence interval for ΔROC AUC included zero (0.0054, 95% CI −0.0060 to 0.0153). Hypertension status provided negligible incremental benefit. Accordingly, much of the predictive performance was attributable to variables already incorporated into the proprietary Tanita equation, whereas waist circumference provided complementary—but not independently dominant—anthropometric information.

#### 3.2.2. Female-Only Sensitivity Analysis

Because the outcome used sex-specific body-fat thresholds, a female-only sensitivity analysis was performed in the 509 female participants, of whom 406 met the prespecified high-adiposity threshold. Model performance remained strong and broadly consistent with the complete-cohort findings. Random forest achieved the highest discrimination among females (ROC AUC = 0.954; PR AUC = 0.976), whereas gradient boosting achieved the highest accuracy (0.937), balanced accuracy (0.881), specificity (0.786), F1 score (0.961), and MCC (0.799). These findings indicate that the primary performance pattern was not solely an artifact of pooling male and female participants. Male-specific estimates were not considered reliable because only 63 males were included, of whom seven had hypertension. The female-only analysis, therefore, serves as a sensitivity analysis of the dominant sex stratum rather than as evidence of equivalent performance across sexes.

Table 6 demonstrates that the female-only analysis closely mirrored the results obtained for the complete cohort. Random forest achieved the highest ROC AUC (0.954), whereas gradient boosting achieved the highest accuracy (0.937), balanced accuracy (0.881), specificity (0.786), F1 score (0.961), MCC (0.799), and the lowest Brier score (0.057). These findings indicate that the overall performance pattern was preserved after restricting the analysis to female participants.

#### 3.2.3. Performance by Hypertension Status

An additional subgroup analysis evaluated the aggregated out-of-fold predictions of the primary gradient-boosting model separately in normotensive and hypertensive participants (Table 7); the model was not refitted within either subgroup. In the normotensive subgroup, gradient boosting demonstrated strong discrimination (ROC AUC = 0.968, PR AUC = 0.984), with accuracy = 0.927, balanced accuracy = 0.907, sensitivity = 0.962, and specificity = 0.851. In contrast, discrimination was substantially lower in the hypertensive subgroup (ROC AUC = 0.679), despite very high sensitivity (0.989). Specificity (0.400), balanced accuracy (0.694), and MCC (0.496) indicated that apparent performance in hypertensive participants was driven largely by extreme outcome prevalence and sensitivity rather than balanced discrimination. Only 10 hypertensive participants were below the high-adiposity threshold, so specificity-related estimates are imprecise.

#### 3.2.4. Comparison with Simple Anthropometric Benchmarks

Simple anthropometric benchmark models demonstrated substantial discriminatory ability (Table 8). Waist circumference-to-height ratio alone achieved a ROC AUC of 0.925, closely approximating the full logistic-regression model (ROC AUC = 0.924), although its balanced accuracy, specificity, MCC, and Brier score were less favorable. Waist circumference alone and body weight plus waist circumference showed lower discrimination, with ROC AUC values of 0.896 and 0.894, respectively. Random forest achieved the highest discrimination (ROC AUC = 0.959), whereas gradient boosting achieved the highest accuracy (0.937), balanced accuracy (0.895), specificity (0.817), F1 score (0.960), MCC (0.817), and the lowest Brier score (0.057). These results indicate that waist circumference-to-height ratio captures much of the available discrimination, while the ensemble models provide modest improvements in threshold-dependent error balance and overall probabilistic accuracy. They do not establish superior probability calibration because calibration remained model-dependent and gradient boosting was overconfident.

Simple anthropometric benchmarks demonstrated substantial discriminatory ability. Waist circumference-to-height ratio alone achieved an ROC AUC of 0.925, closely approximating the full logistic-regression model (ROC AUC = 0.924), although its balanced accuracy, specificity, MCC, and Brier score were less favorable. Waist circumference alone and body weight plus waist circumference showed lower discrimination, with ROC AUC values of 0.896 and 0.894, respectively. Among the machine-learning models, random forest achieved the highest discrimination (ROC AUC = 0.959; PR AUC = 0.980), whereas gradient boosting provided the strongest threshold-dependent classification profile and lowest Brier score. The ensemble models, therefore, offered modest improvements in error balance and overall probabilistic accuracy beyond the simpler benchmarks, but the gradient boosting’s calibration slope of 0.462 shows that a lower Brier score should not be interpreted as well-calibrated absolute probabilities.

**Figure 2:**
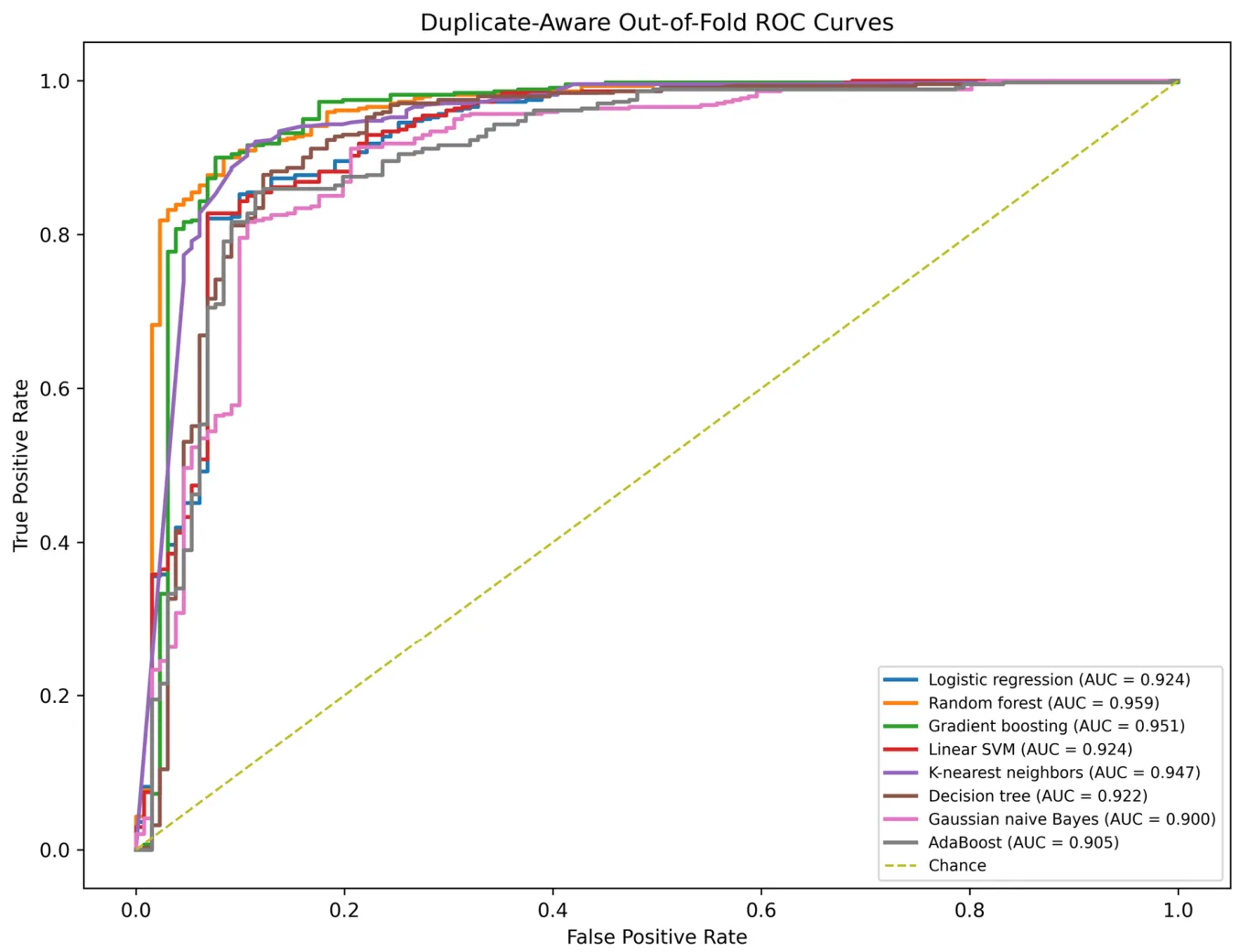
Duplicate-aware out-of-fold receiver operating characteristic curves for the eight algorithms. Values in parentheses are ROC AUC estimates.

Figure 2 visually confirms that random forest provided the highest overall discrimination, with gradient boosting and k-nearest neighbors following closely. The curves for the leading models clustered near the upper-left corner, indicating high sensitivity across relatively low false-positive rates. Their substantial overlap, together with the overlapping confidence intervals in Table 2, suggests that the numerical ranking among the top models should not be interpreted as definitive superiority. Gaussian naive Bayes and AdaBoost showed comparatively weaker global discrimination.

**Figure 3:**
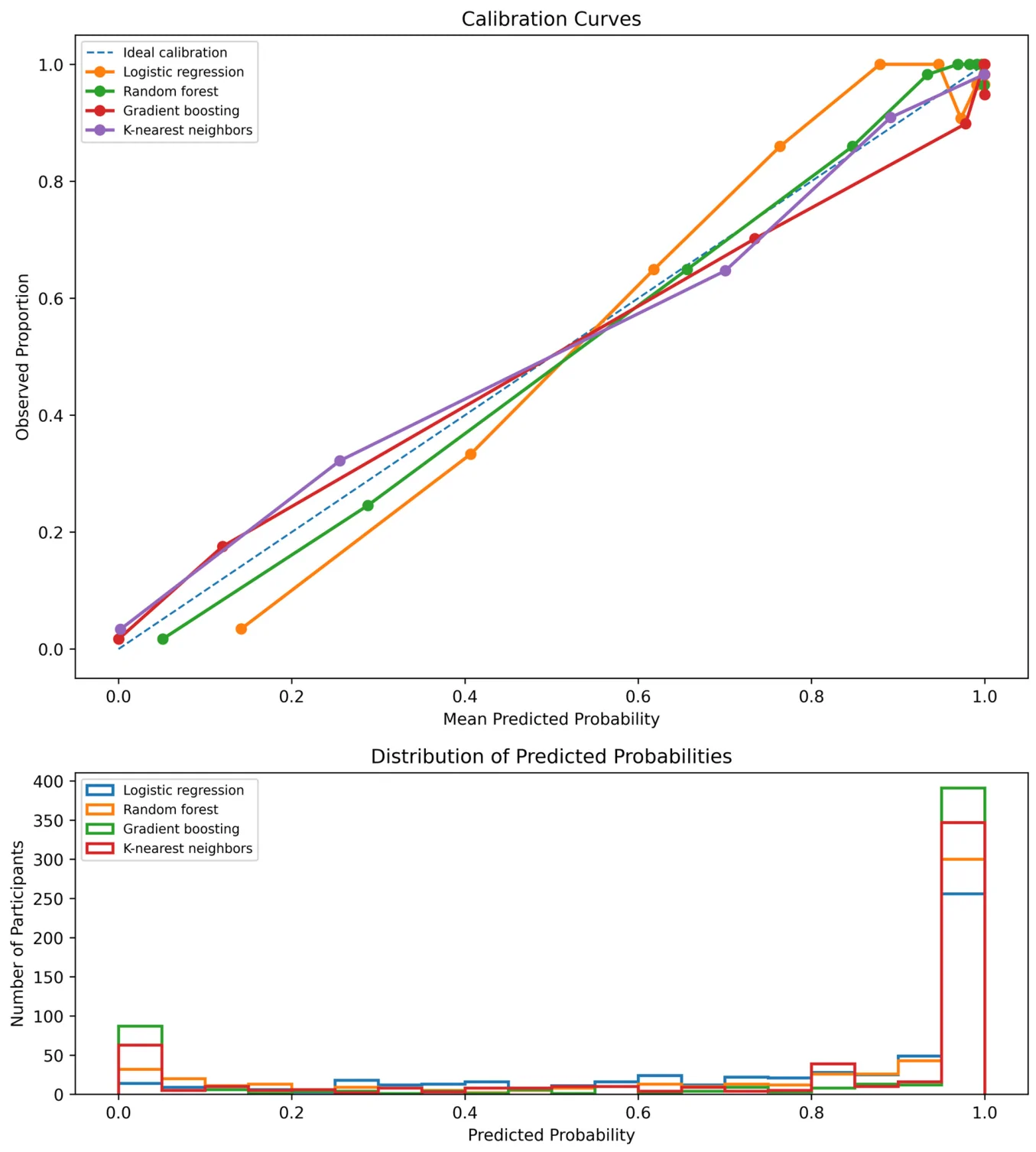
Calibration assessment for logistic regression, random forest, gradient boosting, and k-nearest neighbors. The upper panel presents calibration curves against the ideal calibration line, and the lower panel shows the distribution of duplicate-aware out-of-fold predicted probabilities. Gradient boosting showed overconfident probabilities, consistent with its calibration slope of 0.462.

Figure 3 illustrates that logistic regression and random forest tracked the ideal calibration line more closely across the central probability range than gradient boosting and k-nearest neighbors. The larger deviations of gradient boosting and k-nearest neighbors are consistent with their calibration slopes below one and indicate that their predicted probabilities were too extreme. The plot, therefore, reinforces that strong discrimination and threshold-based accuracy do not necessarily imply reliable absolute probabilities. Estimates at the probability extremes should be interpreted cautiously because calibration bins may contain fewer observations.

**Figure 4:**
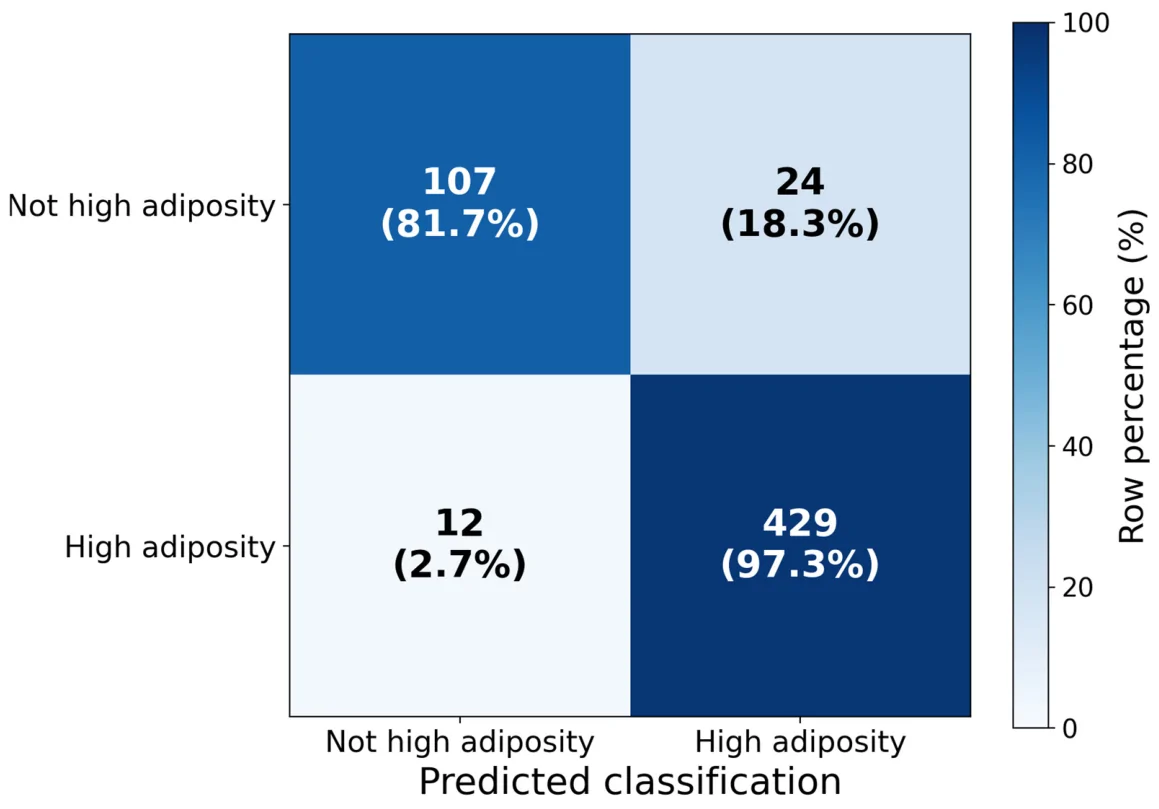
Duplicate-aware out-of-fold confusion matrix for gradient boosting at the prespecified 0.50 threshold. Cells show counts and row percentages.

The confusion matrix was derived from the aggregated out-of-fold predictions generated by repeated duplicate-aware group cross-validation. At the prespecified threshold of 0.50, gradient boosting correctly classified 429 of 441 participants with the Tanita BIA-defined high-adiposity phenotype (97.3%) and 107 of 131 participants below the threshold (81.7%), yielding 536 correct classifications among 572 participants (accuracy = 93.7%). The model produced 12 false-negative (2.7%) and 24 false-positive (18.3%) classifications (Figure 4).

### 3.3. Calibration and Feature Interpretation

Calibration differed substantially despite strong discrimination. Logistic regression had a calibration intercept of −0.024 and slope of 1.050, while random forest had an intercept of −0.093 and slope of 1.127. Gradient boosting had an intercept of 0.145 and a slope of 0.462, indicating that its probabilities were too extreme, even though its threshold-based classifications were strong. This distinction supports reporting both calibration and discrimination rather than selecting a model from accuracy alone.

A SHAP analysis of the final gradient-boosting model identified waist circumference as the dominant contributor, followed by body weight and height. Higher waist circumference and body weight generally shifted the predictions toward the high-adiposity class. Hypertension status had a smaller contribution, and sex contributed minimally after the sex-specific outcome definition and the other anthropometric predictors were considered.

**Figure 5:**
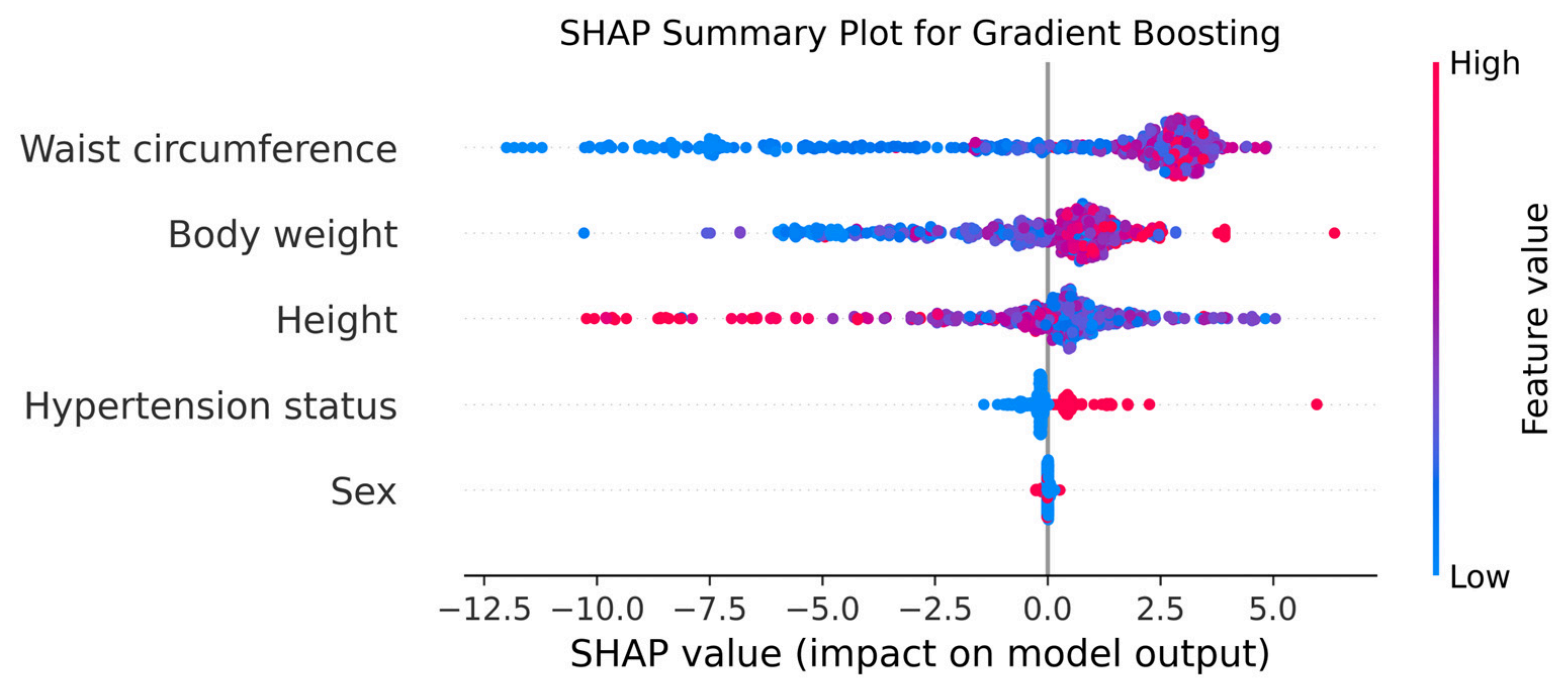
SHAP summary plot for the final gradient-boosting model fitted to the full analytic dataset. Positive SHAP values shift predictions toward the high-adiposity class.

Figure 5 shows both the direction and participant-level variability of the feature contributions. Higher waist circumference and body weight values generally shifted predictions toward the high-adiposity class, whereas lower values shifted predictions in the opposite direction. Waist circumference also displayed the widest SHAP range, indicating the greatest individual-level influence. Height showed a more heterogeneous and generally inverse contribution after the other variables were considered. Hypertension status produced a smaller positive shift for many participants, while sex contributed minimally. The dispersion of points indicates that the effect of a feature was not identical across participants.

**Figure 6:**
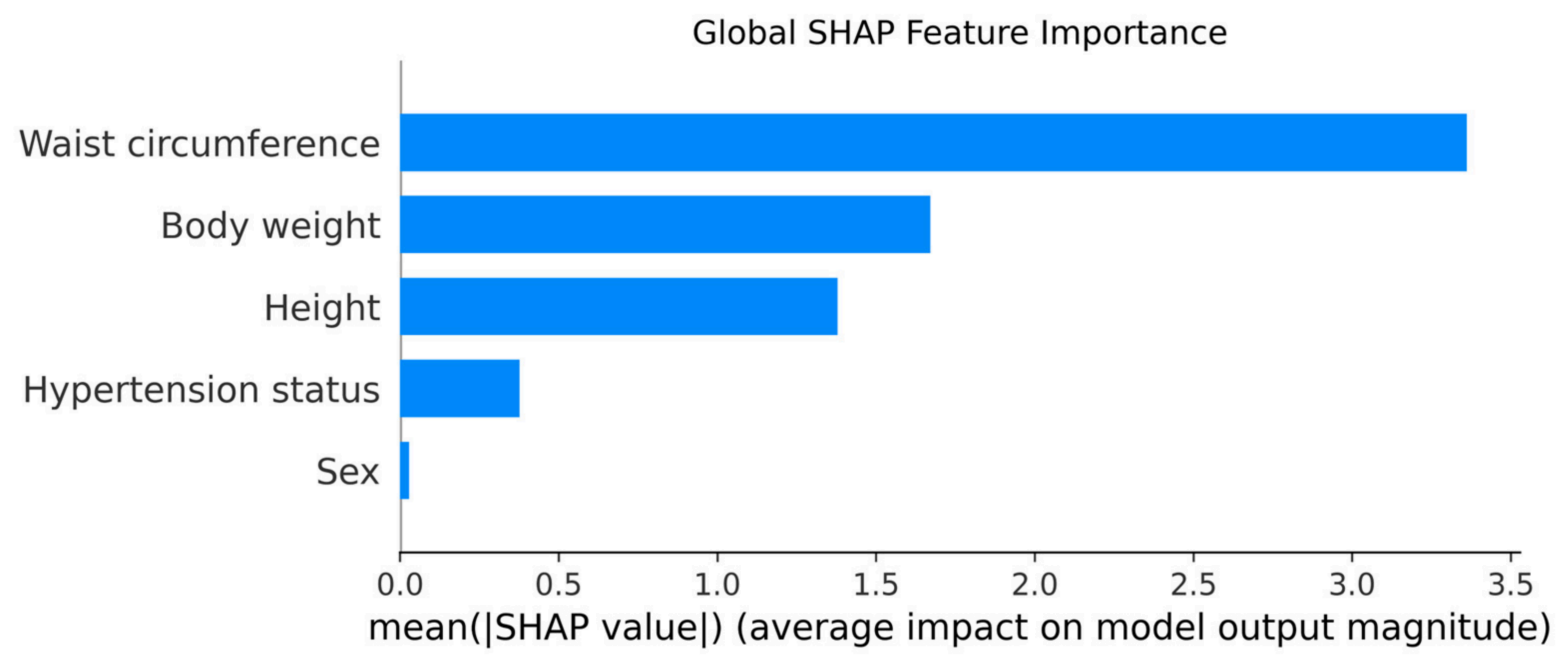
Mean absolute SHAP values showing global feature importance for the final gradient-boosting model.

Figure 6 quantifies the global ranking observed in the SHAP summary plot. Waist circumference had the largest mean absolute SHAP value (3.36), approximately twice that of body weight (1.67), followed by height (1.38), hypertension status (0.38), and sex (0.03). The model, therefore, relied predominantly on anthropometric information, particularly waist circumference, whereas hypertension status and sex added comparatively little incremental information. The dominance of waist circumference in the SHAP analysis should not be interpreted as evidence that it independently generated most of the model’s discrimination. SHAP values describe feature contributions within the fitted complete model, whereas the ablation analysis showed that sex, height, and body weight already produced strong discrimination before waist circumference was added. The two analyses, therefore, address different questions and should be interpreted together. Taken together, the SHAP and ablation analyses suggest that waist circumference contributes complementary anthropometric information beyond the variables incorporated into the proprietary BIA equation rather than acting as the sole driver of model discrimination. These values describe model-specific predictive contributions and should not be interpreted as causal effects. Because waist circumference, body weight, and height are moderately correlated anthropometric variables, SHAP feature importance should be interpreted cautiously. Correlated predictors can share predictive information and redistribute attribution among features without materially changing the overall model performance. Consequently, the ranking of SHAP values should not be interpreted as a definitive measure of independent biological importance.

## 4. Discussion

This study re-evaluated the anthropometric classification of a prespecified BIA-derived high-adiposity phenotype using a validation strategy that kept identical predictor profiles within the same fold. Three findings are central. First, random forest provided the highest discrimination, whereas gradient boosting produced the strongest threshold-based classification and the lowest Brier score. Second, calibration materially changed the model interpretation: gradient boosting and k-nearest neighbors generated overly extreme probabilities, while logistic regression and random forest were better calibrated. Third, waist circumference was the dominant explanatory feature, followed by body weight and height. Together, these findings support the predictive value of simple anthropometry but also show why discrimination, calibration, data integrity, and outcome definition must be considered jointly.

The additional ablation analysis clarified the potential magnitude of partial incorporation bias. A logistic-regression model containing only sex, height, and body weight, all of which are entered into the Tanita device, already achieved strong discrimination (ROC AUC = 0.920). Thus, a substantial proportion of the observed discrimination can be explained by variables that contribute directly to the device-derived reference outcome. Waist circumference increased ROC AUC only modestly, although it produced a more consistent improvement in the Brier score and improved specificity from 0.649 to 0.695. Hypertension status provided virtually no incremental predictive value after the anthropometric variables were included. Consequently, the high performance of the complete models should not be interpreted as an independent prediction of biological adiposity. Rather, it reflects the classification of a device-defined phenotype, with some additional information contributed by waist circumference.

These findings also explain why logistic regression performed competitively despite its comparatively simple structure because much of the predictive information was already contained within variables directly used by the reference device.

The female-only sensitivity analysis further addressed the use of sex-specific outcome thresholds. Performance remained strong among the 509 female participants, with random forest achieving an ROC AUC of 0.954 and gradient boosting maintaining a strong threshold-based performance. This suggests that the principal findings were not solely produced by pooling the participants across sexes. Nevertheless, the study population was markedly imbalanced by sex. Only 63 males were available, including only seven males with hypertension, making male-specific estimates highly unstable. The present findings should, therefore, not be interpreted as establishing equivalent model performance in males and females, and adequately powered sex-balanced external validation is required. Sex-related heterogeneity is biologically plausible because total and regional adiposity and blood-pressure trajectories differ between women and men [5,6]. However, menopausal status, sex-hormone measures, and other endocrine variables were unavailable, so the present analysis cannot identify hormonal mechanisms.

Although random forest achieved the highest discrimination, gradient boosting provided the best overall balance between discrimination, threshold-based classification performance, and probabilistic accuracy, as reflected by its superior balanced accuracy, MCC, and lowest Brier score.

The hypertension-stratified analysis further demonstrated that pooled performance concealed substantial subgroup differences. Gradient boosting performed well among normotensive participants, with an ROC AUC of 0.968, balanced accuracy of 0.907, specificity of 0.851, and MCC of 0.829. In contrast, among hypertensive participants, the ROC AUC was 0.679, specificity was 0.400, and MCC was 0.496, despite an accuracy of 0.957 and F1 score of 0.978. This apparent discrepancy reflects the extreme outcome prevalence in the hypertensive stratum, in which 177 of 187 participants met the high-adiposity definition. High accuracy and F1 score in this subgroup should, therefore, not be interpreted as evidence of balanced or clinically reliable classification. These findings also support the ablation analysis showing that hypertension status contributed negligible incremental information beyond anthropometric variables. Interpretation remains cautious because only 10 hypertensive participants were below the high-adiposity threshold, resulting in limited precision for specificity-related estimates.

The benchmark analysis provides additional clinical context. Waist circumference-to-height ratio alone achieved a discrimination comparable to the full logistic-regression model, suggesting that a simple anthropometric ratio captures a substantial proportion of the available signal. However, the ensemble models provided better balanced accuracy, specificity, MCC, and Brier scores. Thus, the results do not establish that complex machine-learning methods are clinically necessary, but they do show modest improvements in error balance and probabilistic performance beyond the simpler benchmarks. The principal contribution of the study, therefore, remains the rigorous validation framework rather than a claim that algorithmic complexity is inherently superior.

The duplicate-aware validation is an important methodological contribution. In the original record-level split, 26 of 115 test observations had an identical predictor/outcome profile in the training set. Dependence between the development and validation observations can produce substantial optimism, particularly in modest datasets [12]. Assigning all identical profiles to the same fold does not establish that repeated rows represented the same participant; some may have been distinct individuals with rounded measurements. It nevertheless provides a conservative safeguard when unique identifiers are unavailable. Repeated group cross-validation also uses the available sample more efficiently than a single split, although it does not remove model-development uncertainty or replace an independently collected validation cohort [16,26].

The head-to-head sensitivity analysis further showed that the effect of duplicate-aware grouping was model dependent. The clearest optimism under conventional record-level validation occurred for gradient boosting and random forest, whereas differences were negligible for the linear models and Gaussian naive Bayes. This pattern is plausible because flexible tree-based learners are better able to exploit exact or near-exact predictor configurations. However, grouping did not uniformly reduce every metric for every algorithm; small reversals were observed for some models, likely reflecting fold composition and sampling variability. The purpose of duplicate-aware validation was, therefore, not to guarantee lower estimates in every comparison, but to remove a specific pathway through which identical predictor profiles could be represented in both the training and validation data.

An additional limitation is that age could not be included in the analysis because it was unavailable in the source dataset. Since age contributes to the proprietary BIA equation, the present models cannot quantify the incremental value of age or separate its contribution from that embedded within the device-derived reference outcome.

The results can be interpreted in relation to recent obesity-classification studies. Yáñez-Sepúlveda et al. evaluated six algorithms in 5372 adults and reported that random forest achieved the best overall performance, including an AUC of 0.947 [10]. Their strongest features were fat mass index, fat-free mass index, and BMI, all of which contain substantial body-composition information. Nematollahi et al. likewise showed that body-composition measures can support accurate hypertension status classification [11]. The present findings are directionally consistent with the strong performance of tree-based methods, but the predictor design is deliberately more restrictive because BMI, body-fat percentage, and BIA-derived mass indices were excluded. This distinction reduces direct circularity, although it does not eliminate incorporation between the predictors and the proprietary BIA equation used to generate the outcome.

Other recent models further demonstrate that body fat can be approximated from accessible body measurements, while also illustrating why performance estimates are not directly interchangeable. Xu et al. developed and validated equations for DXA-derived body-fat percentage using BMI and sociodemographic variables in nationally representative US samples, reporting coefficients of determination of approximately 0.87 [27]. Jeon et al. used three-dimensional body-scan measurements in 160 Korean adults and reported higher test-set classification performance than BMI or BIA in a 40-person test sample [28]. These studies address different outcomes, populations, measurement technologies, and validation structures. Their results support the broader feasibility of anthropometric prediction, but they also emphasize that high performance may depend strongly on the information content of the predictors, the reference method, and population-specific model development.

The distinction between discrimination and calibration is clinically consequential. Random forest achieved the highest ROC AUC and a calibration slope close to one, whereas gradient boosting yielded stronger F1 and Matthews correlation coefficient values, but a calibration slope of 0.462. A model may rank participants correctly while assigning probabilities that are systematically too extreme [17]. Such a model may appear effective at a fixed threshold yet provide misleading absolute risk estimates. Before probability-based clinical use, the model would require evaluation in an independent cohort and, if necessary, recalibration. For a binary screening application, the threshold should be prespecified according to the intended pathway, and the consequences of false-positive and false-negative classifications should be evaluated using decision-analytic methods.

The dominant contribution of waist circumference within the fitted model is biologically and clinically plausible. Waist circumference captures central body size and correlates with abdominal and visceral adiposity, which contribute to cardiometabolic risk. The large DXA-based analysis by Jeong et al. showed that BMI–body-fat relationships vary across demographic groups [3], whereas the UK Biobank analysis by Oguntade et al. demonstrated that central adiposity provides prognostic information beyond general adiposity for incident heart failure [4]. In the present benchmark analysis, however, waist circumference-to-height ratio alone achieved a discrimination comparable to the complete logistic-regression model. Thus, the findings do not demonstrate that a complex algorithm is clinically necessary. Future external studies should retain these simple comparators, evaluate clinically prespecified cut points and parsimonious penalized regression, and compare the net benefit rather than relying on AUC alone.

A central limitation is partial incorporation between the predictors and the reference outcome. Proprietary BIA equations commonly combine impedance with sex, age, height, and body weight. Consequently, predicting BIA-derived body fat from sex, height, and body weight may partly reconstruct the device equation, even when the body-fat percentage and BMI are excluded. Recent DXA validation studies have shown that BIA performance is equation- and population-dependent [7,8,9]. Acute fluid intake can also alter BIA-derived body-composition estimates, underscoring the importance of standardized premeasurement conditions [29]. In adults with overweight or obesity, Masset et al. found that several existing equations lacked validity and that population-specific equations improved agreement with DXA [30]. These data reinforce the need to interpret the present outcome as a device-specific phenotype and to validate future models against DXA or a multi-compartment reference method.

Another important limitation is that all algorithms were evaluated using prespecified hyperparameter values rather than nested hyperparameter optimization. This design was intentionally adopted to maintain identical validation conditions across models and to isolate the effect of the duplicate-aware validation strategy rather than differences arising from extensive model tuning. Consequently, the observed ranking among algorithms should be interpreted only within the context of these prespecified model configurations and should not be regarded as definitive evidence that one classifier is intrinsically superior. Alternative hyperparameter optimization strategies, particularly for the ensemble-based algorithms, could modify the relative ranking observed in the present study. Future studies should, therefore, investigate whether the relative ranking observed here remains stable after comprehensive nested hyperparameter optimization.

The threshold definition also requires caution. The 25%/35% cut points are established research conventions [21], but the outcome-based analysis by Potter et al. proposed approximately 30% for males and 42% for females as thresholds corresponding more closely to metabolic-syndrome burden [22]. In addition, the contemporary clinical framework distinguishes excess adiposity from clinical obesity, which requires evidence of organ dysfunction or functional impairment [2]. The present models, therefore, classify a prespecified BIA threshold; they do not diagnose clinical obesity, establish treatment eligibility, or replace a comprehensive clinical assessment.

The study’s principal contribution is methodological rather than a claim of deployment readiness. Transparent data auditing, duplicate-aware validation, training-fold preprocessing, repeated out-of-fold estimation, bootstrap confidence intervals, calibration assessment, and explicit separation of the high-adiposity phenotype from clinical obesity collectively provide a more credible evaluation than a conventional single random split. The persistence of strong discrimination after these safeguards suggests that basic anthropometric variables contain substantial information about the device-defined outcome. At the same time, the calibration differences, incorporation bias, class imbalance, and single-center sampling show that strong internal classification is insufficient evidence for clinical implementation.

The inclusion of hypertension status as a predictor should be interpreted with caution. Because participants were recruited from clinical settings, hypertension may partly reflect characteristics of the outpatient population rather than an entirely independent biological determinant of adiposity. Consequently, hypertension should not be interpreted as a causal predictor of obesity but rather as a clinically relevant cardiometabolic characteristic that may contribute to classification performance. Future studies involving population-based cohorts and external validation datasets are warranted to evaluate the independent predictive value of hypertension status while minimizing potential recruitment-related bias.

### 4.1. Strengths and Limitations

The strengths include the transparent identification of invalid placeholder-coded records, prevention of exact-profile overlap across folds, preprocessing confined to training folds, repeated group cross-validation, out-of-fold probability aggregation, reporting across discrimination, threshold-based classification, calibration, and overall prediction error, bootstrap confidence intervals, and explicit distinction between a device-defined high-adiposity phenotype and clinical obesity. The comparison of eight commonly used algorithms under a common validation framework also reduces the risk that performance differences simply reflect inconsistent preprocessing or data partitioning.

Several limitations remain. The sample was recruited from one clinical center by convenience sampling and was highly imbalanced by sex, hypertension status, and outcome prevalence, limiting transportability and the precision of subgroup estimates. Neither the exact recruitment dates nor confirmation of consecutive enrollment was retained in the de-identified analytic files available for this secondary analysis. Therefore, these details could not be reconstructed. Unique participant identifiers were unavailable, so true duplicate entries could not be distinguished from distinct people with identical rounded measurements. Age was absent from the source analytic files, even though it was entered into the BIA device and may influence both body composition and hypertension status. The foot-to-foot BIA instrument used a proprietary equation and was not validated against DXA or a multi-compartment reference in this sample; raw resistance and reactance values were also unavailable. The 25% and 35% threshold is not universal and does not define clinical obesity. Confidence intervals derived from aggregated out-of-fold predictions do not fully represent uncertainty from model selection and refitting. Hyperparameters were prespecified rather than optimized in nested cross-validation. The study did not include an independent external cohort, temporal or geographic validation, an adequately powered male-specific assessment, formal fairness analysis, recalibration, or decision-curve analysis. Finally, sex, height, and body weight overlap with inputs commonly used by BIA equations, creating partial incorporation and limiting claims that the models predict an independent biological reference.

### 4.2. Future Research

Future studies should prospectively collect unique participant identifiers, exact recruitment dates, age, ethnicity, menopausal status, medication exposure, physical activity, dietary factors, hydration indicators, and raw resistance and reactance measurements. Development cohorts should be sex-balanced, multicenter, and sufficiently heterogeneous for prespecified subgroup assessment. Outcomes should be validated against DXA or, preferably, a multi-compartment body-composition method. Simple comparators—including waist circumference, waist circumference-to-height ratio, prespecified clinical cut points, and parsimonious penalized regression—should be retained alongside machine-learning algorithms. Model tuning must be separated from performance estimation through nested resampling. External studies should evaluate calibration in the large, calibration slope, net benefit, subgroup performance, and model stability, with the sample size determined using contemporary precision-based methods [16,26].

## 5. Conclusions

In conclusion, the evaluated machine-learning models demonstrated strong internal classification of a prespecified Tanita BIA-derived high-adiposity phenotype using readily obtainable anthropometric variables under duplicate-aware internal validation. Random forest showed the highest discrimination, whereas gradient boosting achieved strong fixed-threshold classification performance and the lowest Brier score. However, its calibration slope of 0.462 indicates overconfident probabilities and limits the interpretation of individual predicted probabilities. The ablation analysis showed that much of the discrimination was attributable to sex, height, and body weight, which are incorporated into the proprietary Tanita equation. Meanwhile, waist circumference provided only a small and statistically uncertain incremental increase in ROC AUC, and hypertension status contributed negligibly. Accordingly, the present findings establish internal classification performance for a device-defined outcome only. They do not establish clinical utility, external transportability, or prediction of adiposity against an independent criterion method, such as DXA or MRI. External validation, prospective calibration assessment, and comparison with criterion body-composition methods are required before any broader clinical application can be considered.

## Figures and Tables

**Table 1:** Participant characteristics by hypertension status.

Characteristic	Normotensive (*n* = 385)	Hypertensive (*n* = 187)	SMD
Female sex, *n* (%)	329 (85.5)	180 (96.3)	0.382
High-adiposity phenotype, *n* (%)	264 (68.6)	177 (94.7)	0.715
Height, cm	163.34 ± 8.11	158.17 ± 6.18	−0.717
Body weight, kg	78.99 ± 18.27	93.23 ± 20.25	0.738
BMI, kg/m^2^	29.61 ± 6.50	37.37 ± 8.46	1.028
Waist circumference, cm	96.99 ± 19.26	117.19 ± 16.25	1.133
Body fat, %	36.61 ± 9.19	44.29 ± 7.85	0.899

Values are mean ± SD unless otherwise stated. SMD, standardized mean difference; BMI and body-fat percentage were descriptive variables and were not model predictors.

**Table 2:** Duplicate-aware out-of-fold discrimination and fixed-threshold classification performance.

Model	Discrimination		Fixed-Threshold Classification (0.50)			
	ROC AUC (95% CI)	PR AUC (95% CI)	Balanced Accuracy (95% CI)	Sensitivity (95% CI)	Specificity (95% CI)	F1 (95% CI)
Gradient boosting	0.951 (0.921–0.974)	0.964 (0.942–0.991)	0.895 (0.849–0.920)	0.973 (0.955–0.984)	0.817 (0.733–0.870)	0.960 (0.943–0.969)
Random forest	0.959 (0.933–0.977)	0.980 (0.956–0.993)	0.859 (0.826–0.902)	0.971 (0.948–0.982)	0.748 (0.687–0.832)	0.949 (0.935–0.962)
K-nearest neighbors	0.947 (0.921–0.968)	0.975 (0.962–0.987)	0.846 (0.831–0.905)	0.952 (0.939–0.975)	0.740 (0.710–0.847)	0.939 (0.933–0.961)
Logistic regression	0.924 (0.891–0.952)	0.965 (0.943–0.982)	0.828 (0.783–0.864)	0.961 (0.943–0.977)	0.695 (0.611–0.763)	0.937 (0.922–0.949)
Linear SVM	0.924 (0.887–0.953)	0.965 (0.941–0.983)	0.828 (0.782–0.866)	0.964 (0.946–0.980)	0.687 (0.603–0.763)	0.937 (0.923–0.951)
Decision tree	0.922 (0.898–0.955)	0.949 (0.948–0.985)	0.858 (0.799–0.876)	0.968 (0.939–0.975)	0.748 (0.641–0.786)	0.948 (0.924–0.952)
Gaussian naive Bayes	0.900 (0.865–0.933)	0.954 (0.932–0.974)	0.845 (0.811–0.874)	0.796 (0.753–0.828)	0.893 (0.840–0.947)	0.871 (0.843–0.891)
AdaBoost	0.905 (0.869–0.937)	0.948 (0.920–0.976)	0.747 (0.712–0.801)	0.975 (0.955–0.984)	0.519 (0.458–0.626)	0.921 (0.908–0.935)

CIs were obtained from 2000 stratified bootstrap resamples of aggregated out-of-fold predictions. PR AUC, precision-recall area under the curve.

**Table 3:** Overall performance, probability calibration, and confusion-matrix results.

Model	Overall Performance and Error			Calibration		Confusion Matrix	
	Accuracy (95% CI)	MCC (95% CI)	Brier Score (95% CI)	Calibration Intercept	Calibration Slope	TN/FP	FN/TP
Gradient boosting	0.937 (0.911–0.951)	0.817 (0.738–0.859)	0.057 (0.042–0.073)	0.145	0.462	107/24	12/429
Random forest	0.920 (0.899–0.941)	0.764 (0.699–0.827)	0.058 (0.048–0.072)	−0.093	1.127	98/33	13/428
K-nearest neighbors	0.904 (0.895–0.939)	0.719 (0.694–0.823)	0.063 (0.050–0.078)	0.24	0.315	97/34	21/420
Logistic regression	0.900 (0.876–0.920)	0.704 (0.626–0.765)	0.079 (0.068–0.091)	−0.024	1.05	91/40	17/424
Linear SVM	0.900 (0.878–0.921)	0.704 (0.629–0.771)	0.079 (0.068–0.092)	0.033	0.963	90/41	16/425
Decision tree	0.918 (0.879–0.925)	0.759 (0.643–0.783)	0.071 (0.060–0.090)	0.225	0.756	98/33	14/427
Gaussian naive Bayes	0.818 (0.783–0.844)	0.603 (0.539–0.656)	0.127 (0.108–0.146)	0.801	0.451	117/14	90/351
AdaBoost	0.871 (0.850–0.895)	0.602 (0.528–0.685)	0.109 (0.100–0.118)	−0.488	2.485	68/63	11/430

Ideal calibration intercept = 0 and slope = 1. TN, true negatives; FP, false positives; FN, false negatives; TP, true positives; MCC, Matthews correlation coefficient. Paired bootstrap comparison: adding waist circumference to sex, height, and body weight yielded ΔROC AUC = 0.0054 (95% CI −0.0060 to 0.0153). Changes in F1 score and Brier score are reported descriptively; no inferential claim is made for those metrics.

**Table 4:** Incremental value of waist circumference and hypertension status in duplicate-aware logistic-regression models.

Predictor Set	ROC AUC	PR AUC	Accuracy	Balanced Accuracy	Sensitivity	Specificity	Precision	F1 Score	MCC	Brier Score
Sex + height + body weight	0.920	0.965	0.885	0.802	0.955	0.649	0.901	0.927	0.655	0.086
+Waist circumference	0.925	0.965	0.900	0.828	0.961	0.695	0.914	0.937	0.704	0.079
+Hypertension status	0.924	0.965	0.900	0.828	0.961	0.695	0.914	0.937	0.704	0.079

**Table 5:** Comparison of conventional record-level and duplicate-aware grouped cross-validation.

Model	Naive ROC AUC	Grouped ROC AUC	ΔROC AUC	Naive F1	Grouped F1	ΔF1	Naive Brier	Grouped Brier	ΔBrier
Logistic regression	0.924	0.924	0.000	0.937	0.937	0.000	0.079	0.079	0.000
Random forest	0.962	0.959	−0.003	0.954	0.949	−0.005	0.055	0.058	+0.003
Gradient boosting	0.959	0.951	−0.008	0.966	0.960	−0.007	0.044	0.057	+0.013
Linear SVM	0.924	0.924	0.000	0.937	0.937	0.000	0.079	0.079	0.000
K-nearest neighbors	0.950	0.947	−0.003	0.947	0.939	−0.009	0.060	0.063	+0.003
Decision tree	0.922	0.922	0.000	0.947	0.948	+0.001	0.071	0.071	0.000
Gaussian naive Bayes	0.900	0.900	−0.001	0.871	0.871	0.000	0.127	0.127	0.000
AdaBoost	0.908	0.905	−0.003	0.925	0.921	−0.004	0.108	0.109	+0.001

Δ values were calculated as duplicate-aware grouped cross-validation minus conventional record-level cross-validation. Positive ΔBrier values indicate worse probabilistic accuracy under grouped validation. ROC AUC, receiver operating characteristic area under the curve; F1, F1 score.

**Table 6:** Female-only duplicate-aware out-of-fold performance.

Model	ROC AUC	PR AUC	Accuracy	Balanced Accuracy	Sensitivity	Specificity	Precision	F1 Score	MCC	Brier Score
Logistic regression	0.906	0.964	0.910	0.813	0.975	0.650	0.917	0.945	0.702	0.079
Random forest	0.954	0.976	0.925	0.845	0.980	0.709	0.930	0.954	0.757	0.058
Gradient boosting	0.945	0.967	0.937	0.881	0.975	0.786	0.947	0.961	0.799	0.057
Linear SVM	0.903	0.962	0.908	0.804	0.978	0.631	0.913	0.944	0.694	0.079
K-nearest neighbors	0.941	0.976	0.919	0.852	0.966	0.738	0.936	0.950	0.741	0.063
Decision tree	0.905	0.956	0.916	0.835	0.970	0.699	0.927	0.948	0.725	0.071
Gaussian naive Bayes	0.893	0.953	0.815	0.834	0.803	0.864	0.959	0.874	0.569	0.127
AdaBoost	0.915	0.957	0.894	0.785	0.968	0.602	0.906	0.936	0.646	0.109

**Table 7:** Gradient-boosting performance metrics stratified by hypertension status.

Stratum	*n*	High-Adiposity Phenotype, *n*	ROC AUC	PR AUC	Accuracy	Balanced Accuracy	Sensitivity	Specificity	Precision	F1 Score	MCC	Brier Score
Normotensive	385	264	0.968	0.984	0.927	0.907	0.962	0.851	0.934	0.948	0.829	0.064
Hypertensive	187	177	0.679	0.944	0.957	0.694	0.989	0.400	0.967	0.978	0.496	0.042

Abbreviations: ROC AUC, area under the receiver operating characteristic curve; PR AUC, area under the precision–recall curve; MCC, Matthews correlation coefficient. Performance metrics were calculated from the aggregated out-of-fold predictions of the primary duplicate-aware validation procedure.

**Table 8:** Comparison of simple anthropometric benchmark models and duplicate-aware machine-learning models.

Model	ROC AUC	PR AUC	Accuracy	Balanced Accuracy	Sensitivity	Specificity	Precision	F1 Score	MCC	Brier Score
Waist circumference only	0.896	0.958	0.867	0.766	0.952	0.580	0.884	0.917	0.596	0.107
Waist circumference-to-height ratio only	0.925	0.962	0.893	0.815	0.959	0.672	0.908	0.933	0.682	0.086
Body weight + waist circumference	0.894	0.957	0.871	0.774	0.952	0.595	0.888	0.919	0.608	0.105
Full logistic regression	0.924	0.965	0.900	0.828	0.961	0.695	0.914	0.937	0.704	0.079
Random forest	0.959	0.980	0.920	0.859	0.971	0.748	0.928	0.949	0.764	0.058
Gradient boosting	0.951	0.964	0.937	0.895	0.973	0.817	0.947	0.960	0.817	0.057

## Data Availability

The complete analysis code, model specifications, random seeds, and documentation will be deposited in a public repository upon acceptance. The repository URL and permanent identifier will be added to the final published version. The anonymized dataset will be made available from the corresponding author upon reasonable request, subject to institutional and ethical restrictions.

## Supplementary Materials

The following supporting information can be downloaded at: https://www.mdpi.com/article/10.3390/jcm15166164/s1. Figure S1: Duplicate-aware out-of-fold precision–recall curves for all evaluated classifiers. The horizontal reference line represents the observed high-adiposity prevalence (0.771); Table S1: Invalid placeholder-coded values excluded before analysis.
