# Supplementary material for: Duplicate-Aware Internal Validation of Machine-Learning Models for Classifying a Tanita BIA-Derived High-Adiposity Phenotype Using Simple Anthropometric Predictors
Source: J Clin Med. 2026 Aug 8;15(16):6164. doi: 10.3390/jcm15166164 (PMC13513023; doi:10.3390/jcm15166164)
Supplement: Supplementary file 1 [file jcm-15-06164-s001.zip › jcm-4414677-supplementary.pdf]

Supplementary Material

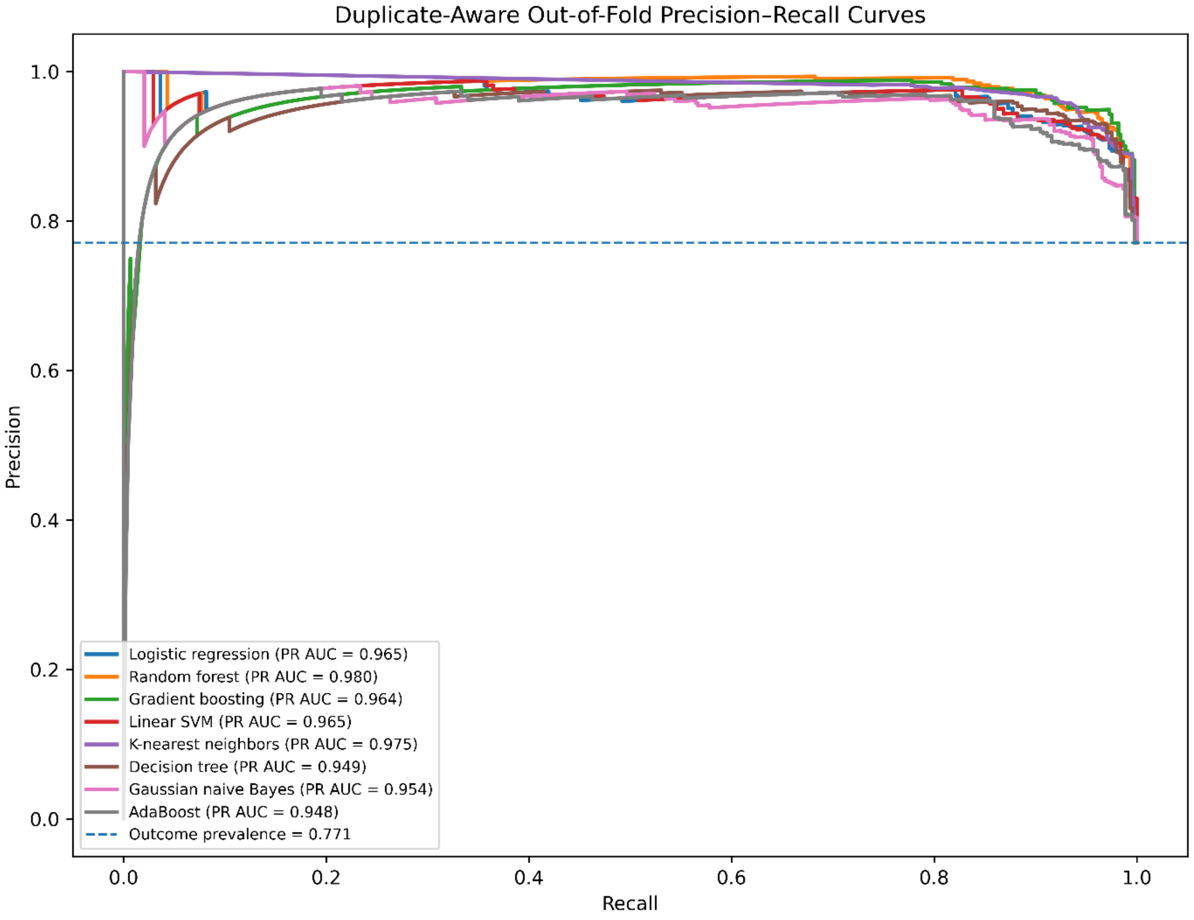

Figure S1. Duplicate-aware out-of-fold precision–recall curves for all evaluated classifiers. The horizontal reference line represents the observed high-adiposity prevalence (0.771).

Table S1. Invalid placeholder-coded values excluded before analysis.

Variable / audit rule	Normotensive	Hypertensive	Total
Body-fat percentage coded as 2	6	4	10
Waist circumference coded as 2	1	0	1
Records excluded (each record counted once)	7	4	11
Records retained for analysis	385	187	572

Note: The audit was conducted on the two prespecified primary data sheets (392 normotensive and 191 hypertensive records). No imputation was performed. The 11 affected records were excluded before model development and validation.
